# Muscarinic M_5_ receptors modulate ethanol seeking in rats

**DOI:** 10.1038/s41386-017-0007-3

**Published:** 2018-02-05

**Authors:** Alice E. Berizzi, Christina J. Perry, David M. Shackleford, Craig W. Lindsley, Carrie K. Jones, Nicola A. Chen, Patrick M. Sexton, Arthur Christopoulos, Christopher J. Langmead, Andrew J. Lawrence

**Affiliations:** 1 Drug Discovery Biology, Monash Institute of Pharmaceutical Sciences, Monash University, Parkville, VIC 3052 Australia; 2 The Florey Institute of Neuroscience and Mental Health, Parkville, VIC 3052 Australia; 3 Centre for Drug Candidate Optimisation, Monash Institute of Pharmaceutical Sciences, Monash University, Parkville, VIC 3052 Australia; 4 Departments of Pharmacology and Chemistry, Vanderbilt Center for Neuroscience Drug Discovery, Vanderbilt University, Nashville, TN 37232 USA; 5 Florey Department of Neuroscience and Mental Health, The University of Melbourne, Parkville, VIC 3010 Australia

## Abstract

Despite the cost to both individual and society, alcohol use disorders (AUDs) remain a major health risk within society, and both relapse and heavy drinking are still poorly controlled with current medications. Here we demonstrate for the first time that a centrally active and selective negative allosteric modulator for the rat M_5_ muscarinic acetylcholine receptor (mAChR), ML375, decreases ethanol self-administration and attenuates cue-induced reinstatement of ethanol seeking in ethanol-preferring (iP) rats. Importantly, ML375 did not affect sucrose self-administration or general locomotor activity indicative of a selective effect on ethanol seeking. Based on the expression profile of M_5_ mAChRs in the brain and the distinct roles different aspects of the dorsal striatum have on long-term and short-term ethanol use, we studied whether intra-striatal microinjection of ML375 modulated ethanol intake in rats. We show in iP rats with an extensive history of ethanol intake that intra-dorsolateral (DL), but not intra-dorsomedial, striatal injections of ML375 reduced ethanol self-administration to a similar extent as the nicotinic acetylcholine receptor ligand varenicline, which has preclinical and clinical efficacy in reducing the reinforcing effects of ethanol. These data implicate the DL striatum as a locus for the effects of cholinergic-acting drugs on ethanol seeking in rats with a history of long-term ethanol use. Accordingly, we demonstrate in rats that selectively targeting the M_5_ mAChR can modulate both voluntary ethanol intake and cue-induced ethanol seeking and thereby provide direct evidence that the M_5_ mAChR is a potential novel target for pharmacotherapies aimed at treating AUDs.

## Introduction

Despite the vast impact alcohol use disorders (AUDs) have within society [1, 2], current pharmacotherapies remain inadequate. Clinically used drugs, such as naltrexone and acamprosate, have been linked with low patient compliance due to adverse side effects, ultimately leading to high relapse rates [3, 4]. Novel pharmacotherapies for the treatment of AUDs may come from a better understanding of the mechanisms that underpin relapse. In this regard, there is a need to understand the underlying neurocircuitry and transmitter/receptor systems that are implicated in the pathophysiology of alcohol abuse to then enable drug discovery programs to identify and validate novel targets.

The M_5_ muscarinic acetylcholine (ACh) receptor (mAChR) is one of five mAChR subtypes and is a G protein-coupled receptor, which couples to G_q/11_ proteins [5, 6]. It has a discrete expression profile being predominantly expressed on dopamine neuron terminals within the dorsal and ventral striatum that potentiate the release of dopamine and glutamate from midbrain projections, and is the sole mAChR expressed postsynaptically on dopaminergic neurons of the ventral tegmental area and substantia nigra pars compacta (SNc) [7, 8]. Furthermore, the M_5_ mAChR is expressed densely in the ventral subiculum, which projects to the NAc shell, an input pathway implicated in context-mediated relapse to ethanol seeking [9, 10]. Accordingly, there is anatomical and neurochemical evidence for a potential role of the M_5_ mAChR in the modulation of reward processing, although this is largely unexplored. Notably, studies in M_5_ mAChR knockout (KO) mice show reduced conditioned place preference and reduced self-administration of cocaine, but not a natural reward [11, 12]. M_5_ mAChR KO mice also demonstrated reduced conditioned place preference to morphine and attenuated morphine withdrawal signs [13]. Taken together, these data support a role of M_5_ mAChRs in modulating the reinforcing effects of drugs of abuse. However, to date, the M_5_ mAChR has not been explicitly studied in models of ethanol use and/or relapse.

Interrogation of the role of M_5_ mAChRs in reward seeking by classical pharmacological techniques remains suboptimal. This is likely because compounds have been traditionally developed to target a highly conserved orthosteric site on the receptor, which means ligands that have been designed to target individual mAChRs often have off-target effects at other mAChR subtypes [14]. However, recent years have seen an increase in the discovery of allosteric ligands that are highly selective for individual mAChR subtypes, which target less well-conserved allosteric site(s) on the receptor and/or exhibit selective cooperativity between receptor and ligand [15]. ML375 has been reported as a selective negative allosteric modulator (NAM) of the M_5_ mAChR [16, 17]. Accordingly, it is now possible to selectively target the M_5_ mAChR in vivo to delineate its functional role(s), which could ultimately aid in the development of refined pharmacotherapies for AUDs and/or other disorders [18, 19].

This study confirms that ML375 is a selective NAM for the rat M_5_ mAChR and can access the brain after systemic administration. We show that systemic ML375 attenuates ethanol self-administration and reduces the ability of drug-associated cues to trigger reinstatement of ethanol seeking, but does not impact sucrose self-administration or general locomotor activity in ethanol-preferring (iP) rats. Furthermore, we show that intra-dorsolateral (DL), but not intra-dorsomedial (DM), striatal injection of ML375 reduced ethanol self-administration to a similar extent as the nicotinic ACh receptor (nAChR) ligand varenicline, which has both preclinical and clinical efficacy in reducing the reinforcing effects of ethanol [20–23] Accordingly, we show for the first time that selectively targeting the M_5_ mAChR can modulate the reinforcing effects of ethanol in rat models of ethanol seeking. We also provide the first direct evidence that the M_5_ mAChR is a potential novel target for pharmacotherapies aimed at treating AUDs.

## Materials and Methods

### Materials

Sources of all materials used are listed in Supplementary information.

### Cell culture

Rat mAChR subtypes (rM_1_–rM_5_; Origene) and human cannabinoid 1 receptor (hCB_1_; ref. 24) constructs were isogenically integrated into FlpIn CHO cells (Invitrogen) and cells were selected in the presence of 600 mg/mL hygromycin B at 37 °C, 5% CO_2_, as previously described for the human M_1_ mAChR [25]. All cells were sub-cultured and seeded as previously described for the CHO-hM_5_ cells [17].

### Radioligand binding

[^3^H]NMS equilibrium binding assays in CHO-rM_1_ to CHO-rM_5_ cells were performed as previously described for CHO-hM_5_ cells [17]. For [^35^S]GTPγS binding, CHO-rM_2_ and CHO-rM_4_ cell membranes were prepared as described in Supplementary information. Membrane [^35^S]GTPγS binding was performed as described in ref 26., except GDP and [^35^S]GTPγS concentrations were 1 μM and 0.3 nM, respectively. [^3^H](+)-pentazocine binding was performed as described in ref. 27.

### Cell-based functional signalling assays

The IP-One assay kit (Cisbio) was used for the direct quantitative measurement of inositol phosphate (IP) accumulation in CHO-rM_1_, CHO-rM_3_ or CHO-rM_5_ cells, as described previously [17]. The CHO-hCB_1_ pERK1/2 assays were performed as described in ref 24.

### Animals for behavioural studies

All studies were undertaken in accordance with the Prevention Cruelty to Animals Act (2004) and carried out within the guidelines of the National Health and Medical Research Council (NHMRC) Code of Practice for the Care and Use of Animals for Experimental Purposes in Australia (2013) and approved by the Florey Animal Ethics Committee. Adult male iP rats (gift from Professor T.K. Li while at Indiana University) were obtained from in-house breeding at ≥8 weeks of age. Rats were paired-housed at a constant temperature of 21 °C and maintained on a 12 h light/dark cycle (lights on at 7.00 a.m.). Post-surgery rats were singly housed. Water and chow were available ad libitum.

### Rat pharmacokinetics

ML375 pharmacokinetics after either intraperitoneal or oral dosing of rats was determined as described in Supplementary information.

### Dosing for operant chamber studies

ML375 was administered orally as an aqueous 30% (w/v) 2-hydroxypropyl-β-cyclodextrin suspension (30 mg/kg; 10 mL/kg) 27 h, 11 h and 3 h prior to testing. For intra-striatal microinjections, varenicline (5.53 nmol/hemisphere), ML375 (105 pmol/hemisphere) and ML380 (165 pmol/hemisphere) were formulated in artificial cerebrospinal fluid with 2% dimethylsulphoxide (v/v) as the vehicle in all cases for microinjection.

### Ethanol self-administration

Male iP rats (*n* = 14) were trained to self-administer 10% (v/v) ethanol via lever presses under FR3 operant conditions for approximately 12 weeks, in the presence of a 1-s light conditioned stimulus (CS+) occurring when the ethanol solution was delivered and an olfactory cue (S+ cue; one drop of vanilla essence, placed directly below the ethanol-paired (active) lever), as previously described [28, 29], followed by surgery and testing. For oral studies, rats had at least three habituation sessions to the gavaging procedure before dosing with ML375 (30 mg/kg) or vehicle. Rats were then given 7 days of ethanol self-administration before receiving the alternate treatment in a counterbalanced manner. For full details refer to Supplementary material.

### Sucrose self-administration

Male iP rats (*n* = 9) were trained to self-administer sucrose (0.05–1% w/v) under FR3 operant conditions for at least 12 weeks, as previously described [30], followed by testing with the same counterbalanced oral regimen of ML375 described above.

### Locomotor activity

Rats (*n* = 6) were habituated to the locomotor room for at least 3 h. In a counterbalanced manner rats received the same oral dosing schedule of vehicle/ML375 as used in the operant studies prior to testing in a 42 cm (length) × 42 cm (width) × 32 cm (height) transparent locomotor cell (Tru Scan Photobeam Arena, E63-10; Coulbourn Instruments) for 60 min under low light conditions. A second cohort of rats (*n* = 9) were tested in a similar manner with vehicle/ML375 at 56.6 mg/kg p.o.

### Cue-induced reinstatement of ethanol seeking

A separate cohort of rats (*n* = 11) underwent 12 weeks of operant ethanol (10% v/v) self-administration followed by extinction training, where lever presses had no programmed consequences. Extinction occurred in the absence of CS+ and S+ cues. After extinction rats were treated orally with ML375 (30 mg/kg) or vehicle under the same regimen as used for self-administration. Subsequently, rats were tested for cue-induced reinstatement by re-introduction of the ethanol-paired CS+ and S+ cues, but in the absence of reward delivery [28, 31]. Rats were then re-extinguished and submitted to a second reinstatement session with the counterbalanced treatment.

### Stereotaxic implantation of cannula into DL and DM striatum

Surgery was performed as previously described [29]; for full details see Supplementary material.

### DL and DM striatum infusions and verification of injection sites

Microinjections were performed as previously described [29]; for full details see Supplementary material.

### Data and statistical analyses

GraphPad Prism version 7.02 (San Diego, CA) was used for all statistical analysis and curve fitting. Analysis of in vitro signalling and radioligand binding data was as described in Supplementary information and ref 17. All data are represented as mean ± s.e.m. Operant data were analysed by repeated measures (RM) two-way analysis of variance (ANOVA) (treatment x lever) with Tukey’s multiple comparisons post hoc analysis using GraphPad Prism. Significance was set at *P* < 0.05, unless otherwise stated. Extinction data are reported as an average of the last three sessions before reinstatement test.

## Results

### Ethanol self-administration

Prior to pharmacological challenges, rats averaged 101 ± 4.61 ethanol lever presses (0.60 ± 0.03 g/kg per session ethanol intake) and 5.4 ± 0.53 water lever presses (0.36 ± 0.04 g/kg per session water intake) per 20 min session, which results in pharmacologically relevant blood ethanol levels [32]. After surgery, rats returned to pre-surgical levels of ethanol responding prior to testing.

### ML375 is a selective NAM of ACh function at the rM_5_ mAChR

**Fig. 1:**
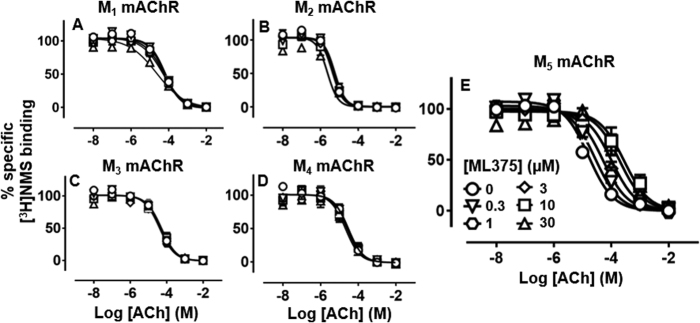
Three-way radioligand binding studies to demonstrate the selective effect of ML375 on ACh-mediated inhibition of [^3^H]NMS binding in whole FlpIn-CHO cells stably expressing rat M_5_ mAChR (**e**) vs. rat M_1_ (**a**), M_2_ (**b**), M_3_ (**c**) and M_4_ mAChR (**d**). Data are expressed as a percentage of specific [^3^H]NMS binding and represent the mean ± S.E.M. of three independent experiments performed in duplicate

**Fig. 2:**
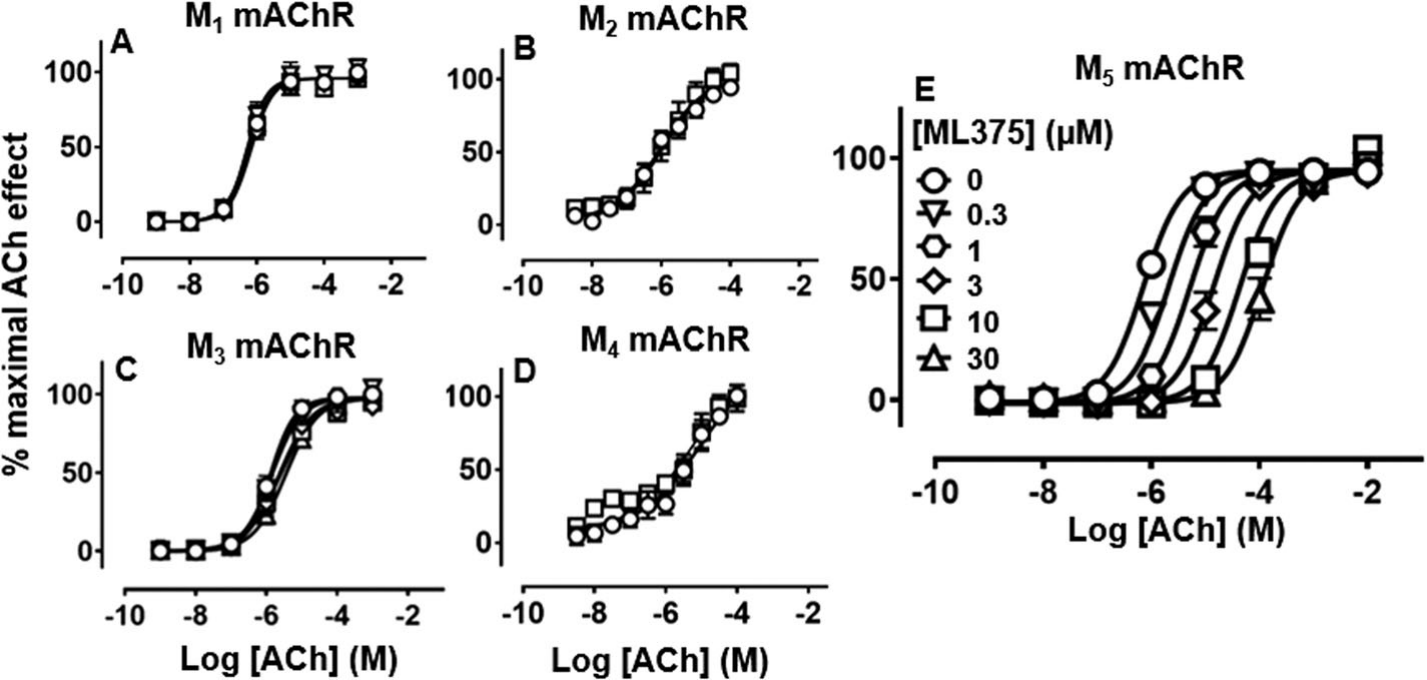
Effect of ML375 on ACh-stimulated IP accumulation in FlpIn-CHO cells stably expressing rat M_1_, M_3_ and M_5_ mAChRs (**a**, **c** and **e**, respectively) and ACh-stimulated GTPγ^35^S binding in membranes from FlpIn-CHO cells stably expressing rat M_2_ and M_4_ mAChRs (**b** and **d**, respectively). Data are expressed as a percentage of the maximal ACh response and represent the mean ± S.E.M. of three to four independent experiments performed in duplicate

Since some allosteric modulators of mAChRs show species differences, we first utilised whole-cell [^3^H]NMS-binding assays to investigate whether ML375 binds in a similar manner to rat orthologues compared to previously reported human mAChR data [16, 17, 33]. ML375 caused a concentration-dependent decrease in the affinity of ACh for the rM_5_ mAChR with neutral-to-weak negative cooperativity with [^3^H]NMS (Fig. 1e). The binding affinity and cooperativity estimates for ML375 (p*K*_B_ = 6.45 ± 0.18; Log *α* = −1.41 ± 0.15) were similar to that previously reported for the hM_5_ mAChR in the same assay (p*K*_B_ = 6.87; Log *α *= −1.37; ref. 17). However, the negative cooperativity with respect to ACh binding was insufficient to account for the higher functional negative cooperativity (Fig. 2e), suggesting that ML375 exerts effects on both ACh affinity and efficacy.

An IP accumulation assay was utilised to investigate the functional interaction of ML375 with ACh at the G_q/11_-linked rM_1_, rM_3_ and rM_5_ mAChRs, and a [^35^S]GTPγS-binding assay was used to assess interactions of ML375 with ACh at the G_i/o_-linked rM_2_ and rM_4_ mAChRs, respectively. ACh-stimulated IP accumulation (rM_1_ pEC_50_ = 6.25 ± 0.04; rM_3_ pEC_50_ = 5.89 ± 0.05; rM_5_ pEC_50_ = 6.15 ± 0.04) and [^35^S]GTPγS-binding assay^35^S binding in transfected CHO cells (rM_2_ pEC_50_ = 6.07 ± 0.24; rM_4_ pEC_50_ = 4.95 ± 0.06; Fig. 2). ML375 caused a parallel rightward shift that did not reach a limit in the ACh concentration–response curve at the rM_5_ mAChR and it showed higher functional affinity (p*K*_B_ = 6.81 ± 0.05; Fig. 2e) than reported for the hM_5_ mAChR (p*K*_B_ = 6.22; ref. 17). This is indicative of high negative cooperativity between ML375 and ACh at the rM_5_ mAChR [17].

ML375 showed high selectivity for the rM_5_ mAChR over other mAChRs, with only weak activity at the rM_3_ mAChR (Fig. 2c) and no significant effects on ACh affinities and potencies at the rM_1_, rM_2_ or rM_4_ mAChRs in either [^3^H]NMS-binding or functional studies (Figs. 1a–d and 2a–d).

Previous studies have suggested M_5_ mAChR-targeting allosteric ligands may show off-target activity at σ1 and cannabinoid CB_1_ receptors [16, 34]. However, ML375 had no apparent effect on either [^3^H](+)-pentazocine binding in mouse brain homogenates (targeting the σ1 receptor) or in in CHO-hCB_1_ ERK1/2 phosphorylation assays at concentrations up to 10 μM (Figure S1).

### ML375 pharmacokinetics

**Fig. 3:**
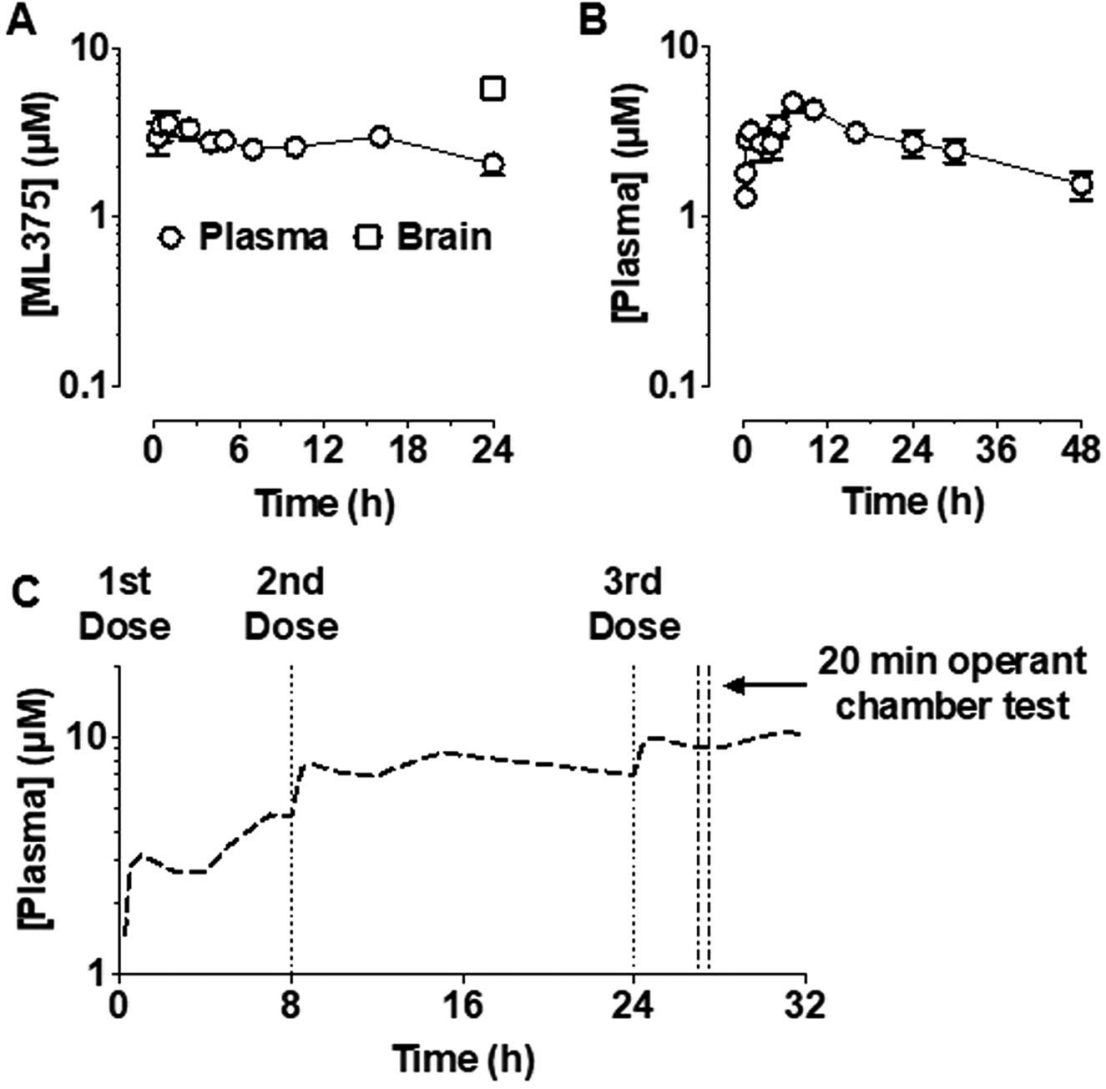
Plasma exposure profile of ML375 after (**a**) 20 mg/kg (intraperitoneal; i.p.) or (**b**) 30 mg/kg (per os; p.o.) dose (*n* = 3). Brain exposure was also measured at 24 h after IP injection. (**c**) Superposition of oral exposure profiles provides a prediction of plasma exposure of ML375 for an operant chamber test session after three doses of ML375, each at 30 mg/kg (p.o.)

Intraperitoneal administration of ML375 (20 mg/kg) in rats yielded an almost flat plasma exposure profile for the compound over a 24 h period (*n* = 3, Fig. 3a), indicating ML375 has a long terminal half-life and low clearance. Furthermore, when measured at the 24 h timepoint, ML375 exhibited a brain-to-plasma ratio of 3.0 ± 0.7. As ML375 solubility is dose limiting and in order to facilitate a potential repeat-dose paradigm, the oral exposure profile of a single, 30 mg/kg dose of ML375 was assessed in rats (*n* = 3). ML375 yielded a *C*_max_ of 4.7 ± 0.7 μM (*T*_max_ = 7 h; Fig. 3b; the initial biphasic nature of the exposure profile likely reflects the early absorption of solubilised compound followed by the delayed dissolution of ML375 in suspension). As it was not possible to increase the dose further, based on superposition of the oral exposure profile, we predicted that a three-dose paradigm should yield sufficient plasma exposure and, by calculation, a sufficient free brain concentration of ML375 to occupy the M_5_ mAChR (based on brain-to-plasma ratio and unbound fraction in brain; Fig. 3c) [16].

### ML375 attenuates ethanol but not sucrose self-administration

**Fig. 4:**
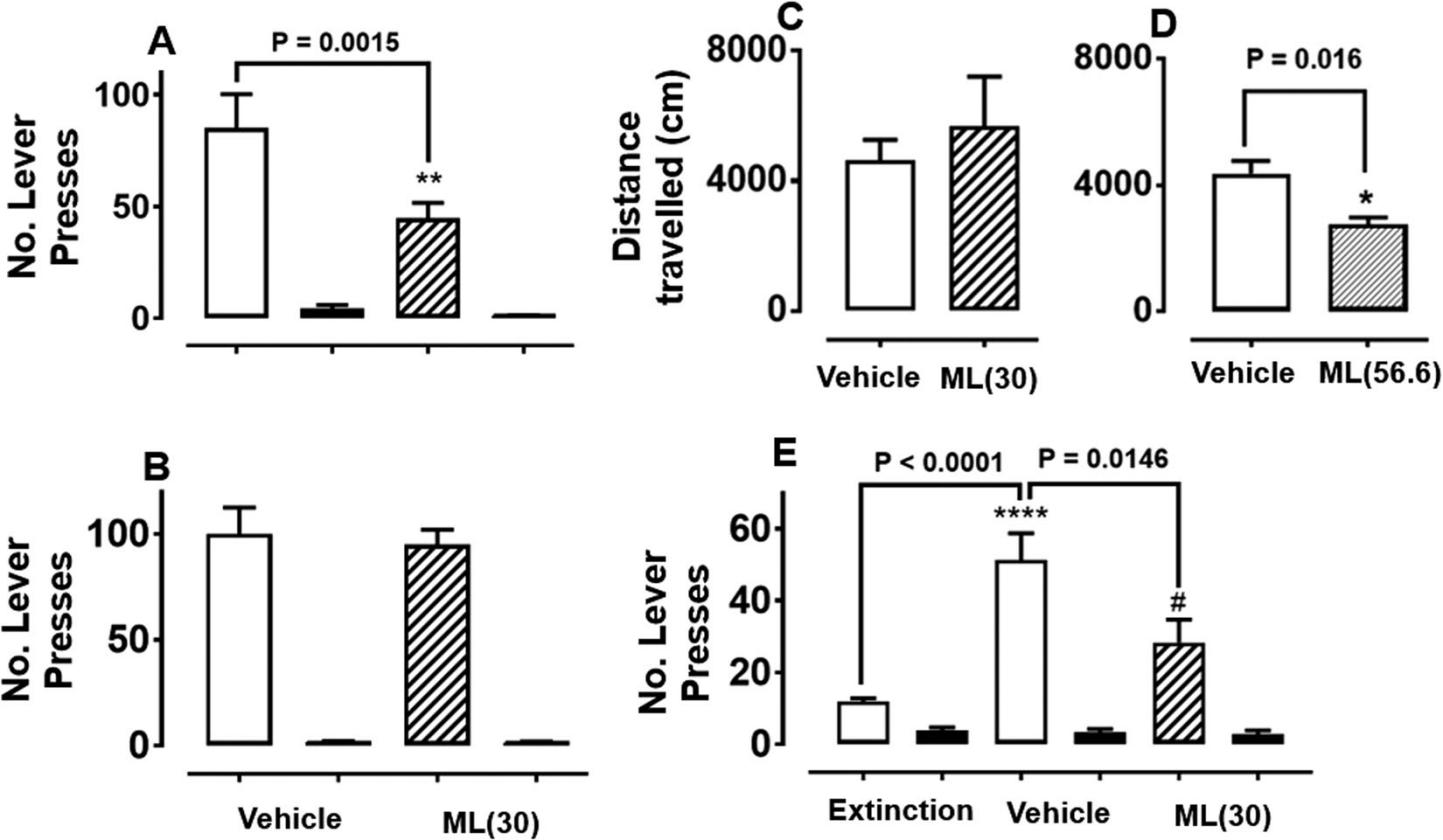
**a**, **b** Self-administration: ML375 significantly reduces operant self-administration of 10% ethanol (**a**; *P* = 0.0015; *n* = 14), but not sucrose (**b**; *P* = 0.96; *n* = 9) or water in iP rats. ML375 was dosed at 30 mg/kg (p.o.) at 27 h, 11 h and 3 h prior to testing. White bars represent ethanol (**a**) and sucrose (**b**) responses following vehicle treatment, respectively; dashed bars represent ethanol (**a**) or sucrose (**b**) responses following ML375 treatment; black bars represent water responses. Data were analysed by repeated measures two-way ANOVA with post hoc Tukey’s multiple comparisons test and expressed as mean ± S.E.M. **P *<* 0.01 denotes significant effect of ML375 treatment on active lever responding for ethanol compared to vehicle. There was no difference in responding for the water-paired lever after ML375 vs. vehicle treatment (*P* = 0.98). **c**, **d** Locomotor activity: There was no significant effect of ML375 (30 mg/kg, p.o.; **c**, dashed bar) compared to vehicle (white bar; *n* = 6) on locomotor activity in iP rats (*P* = 0.53). In contrast, a higher dose of ML375 (56.6 mg/kg, p.o. **d**, dashed bar) significantly reduced locomotor activity compared to vehicle (*n* = 9; *P* = 0.016). Data represent mean ± S.E.M. Data were analysed by repeated measures two-way ANOVA. **e** Cue-induced reinstatement: ML375 attenuates cue-induced reinstatement of ethanol seeking after systemic administration (3 × 30 mg/kg, p.o.; *n* = 11). Data were analysed by repeated measures two-way ANOVA with post hoc Tukey’s multiple comparison test and expressed as mean ± S.E.M. ****P < 0.0001 denotes significant difference of treatment on active lever responding during the reinstatement session as compared to extinction (*P* *<* 0.0001). ^#^*P* < 0.05 denotes significant difference in active lever responding during reinstatement test between ML375 treated rats compared to vehicle (*P* = 0.015). There was no effect on inactive lever responding during cue-induced reinstatement test

Rats were dosed with either ML375 (30 mg/kg; 10 mL/kg) or vehicle at 27 h, 11 h and 3 h prior to test. RM two-way ANOVA revealed significant main effects of treatment (F_(1, 13)_ = 13.2, *P* = 0.003) and lever (F_(1, 13)_ = 39.4, *P* < 0.0001) and a significant treatment x  lever interaction (F_(1, 13)_ = 10.1, *P* = 0.0072) (Fig. 4a). ML375 treatment significantly reduced ethanol responding compared to vehicle (*P* = 0.0015; Tukey’s multiple comparisons). There was no difference in responding for water after ML375 vs. vehicle treatment (*P* = 0.98).

A separate cohort of rats were trained to self-administer sucrose (0.05–1% w/v). Rats underwent an identical dosing regimen of vehicle or ML375 before testing. RM two-way ANOVA revealed a significant main effect of lever (F_(1, 8)_ = 179, *P* < 0.0001) but no effect of treatment (F_(1, 8)_ = 0.13, *P* = 0.73). Accordingly, ML375 did not alter sucrose responding compared to vehicle (*P* = 0.96) and there was also no difference between ML375 and vehicle on water responding (*P* = 1.0; Fig. 4b). These data suggest that systemic administration of ML375 specifically reduces ethanol self-administration in male iP rats.

### ML375 does not affect procedural memory or general locomotor activity

Rats that received ML375 (30 mg/kg, p.o.) demonstrated no difference in latency to first ethanol reward (*P* *=* 0.73; Figure S2), suggesting that ML375 did not cause sedation or a deficit in procedural memory. In a separate cohort of rats there was no difference between vehicle and ML375 (30 mg/kg, p.o.) treatment in locomotor activity (*P* = 0.53; Fig. 4c). Note, however, that a higher dose of ML375 (56.6 mg/kg, p.o.) did reduce locomotor activity (*P* = 0.016; Fig. 4d); hence, all operant studies were limited to 30 mg/kg p.o.

### ML375 attenuates cue-induced reinstatement of ethanol seeking

Subsequently, we examined whether ML375 would affect cue-induced reinstatement of ethanol seeking in a separate cohort of rats. Following extinction rats were dosed with either vehicle or ML375 and underwent a cue-induced reinstatement session. RM two-way ANOVA revealed significant main effects of treatment (F_(2, 20)_ = 8.85, *P* = 0.0018) and lever (F_(1, 10)_ = 144, *P* < 0.0001) and a significant treatment x lever interaction (F_(2, 20)_ = 10.5, *P* = 0.0008). The return of S+ and CS+ cues induced a robust reinstatement of ethanol seeking in vehicle-treated rats (extinction vs. vehicle, *P* < 0.0001, Tukey’s multiple comparisons; Fig. 4e), while ML375 treatment significantly attenuated the reinstatement of ethanol seeking (vehicle vs. ML375, *P* = 0.015; extinction vs. ML375, *P* = 0.13). There were no differences in responding for the previously water-paired lever following vehicle vs. ML375 treatment (*P* > 0.99).

### Microinjection of ML375 in the DL striatum but not DM striatum decreases ethanol self-administration

**Fig. 5:**
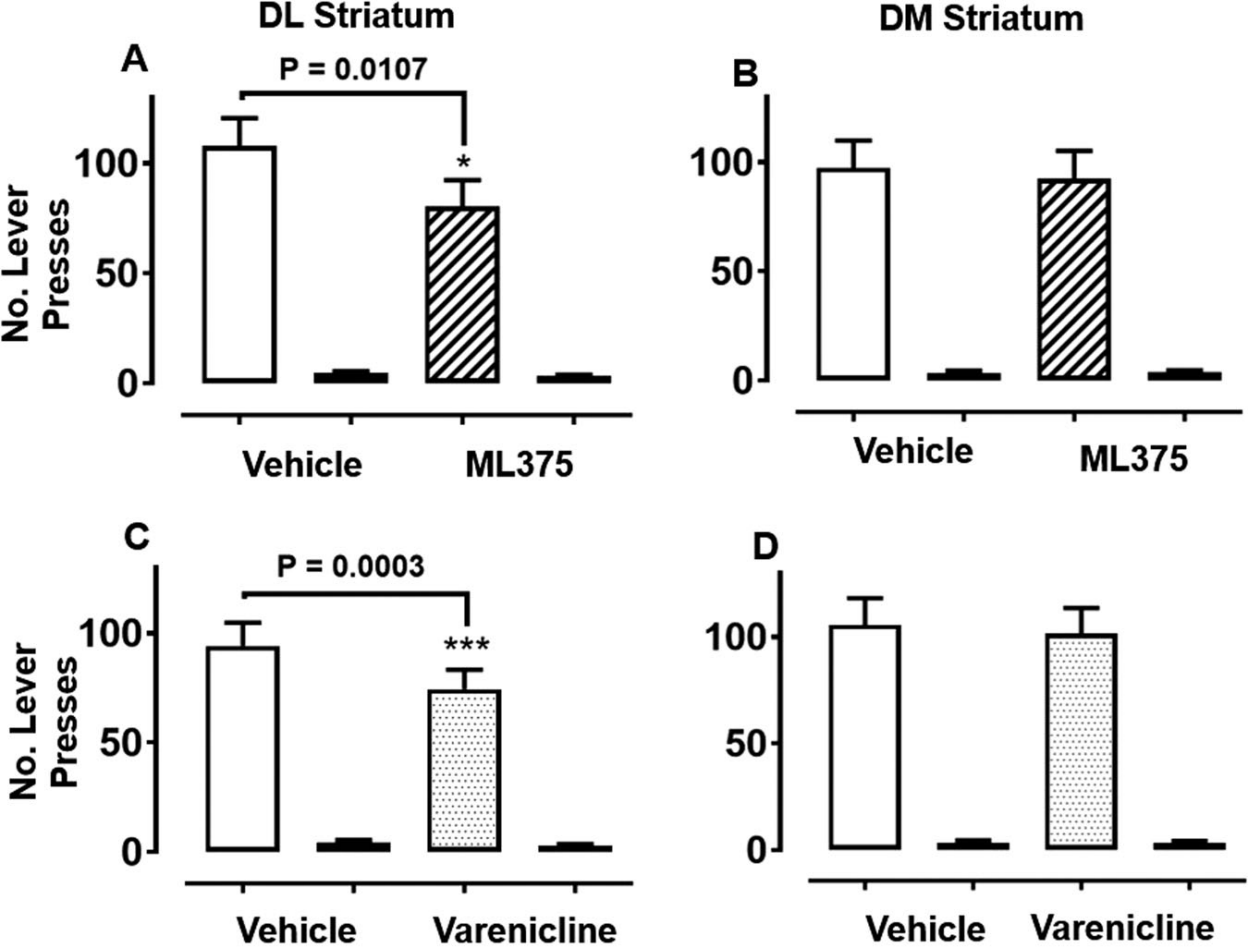
Bilateral infusion of either the M_5_ mAChR NAM ML375 (105 pmol/hemisphere; *P* = 0.011; *n* = 12–16; dashed bars) or the nAChR partial agonist varenicline (5.53 nmol/hemisphere; *P* = 0.0003; *n* = 11–16; dotted bars) into dorsolateral (DL) striatum (**a**, **c**), but not into the dorsomedial (DM) striatum (**b**; *P* = 0.89, **d**; *P* = 0.96) significantly reduces operant self-administration of 10% ethanol in iP rats. White bars represent ethanol responses following vehicle; dashed bars represent ethanol responses following ML375 treatment (**a**, **b**); dotted bars represent ethanol responses following varenicline treatment (**c**, **d**); black bars represent water responses. Data were analysed by repeated measures two-way ANOVA with post hoc Tukey’s multiple comparisons test and expressed as mean ± S.E.M. *P < 0.05 and ***P < 0.001 denotes significant difference of treatment on active lever responding compared to vehicle. There was no effect on water lever presses

To investigate an anatomic locus, ethanol-experienced rats received microinjection of ML375 (105 pmol/hemisphere) and vehicle in a counterbalanced manner into the DL striatum. RM two-way ANOVA revealed significant main effects of both treatment (F_(1, 11)_ = 9.82, *P* = 0.0095) and lever (F_(1, 11)_ = 62.9, *P* < 0.0001) and a significant treatment x lever interaction (F_(1, 11)_ = 7.03, *P* = 0.023) (Fig. 5a). Bilateral intra-DL striatal microinjection of ML375 significantly reduced responding for ethanol compared to vehicle (*P* = 0.011). There was no difference in responding for water after vehicle vs. ML375 microinjection (*P* > 0.99).

A separate cohort of rats received microinjection of ML375 (105 pmol/hemisphere) and vehicle into the DM striatum. There was a main effect of lever (F_(1, 15)_ = 59.4, *P* < 0.0001) but no effect of treatment (F_(1, 15)_ = 0.22, *P* = 0.65) (Fig. 5b). Thus, ML375 injection into the DM striatum of iP rats did not affect ethanol responding. Following experimentation, microinjection sites of ML375 into the striatum of all rats were validated histologically (Figure S3A).

M_5_ mAChRs may functionally interact with α4β2-containing nicotinic receptors [35]. For comparison therefore, we assessed the clinically approved nicotinic receptor partial agonist, varenicline. A cohort of rats received bilateral microinjection of varenicline (5.53 nmol/side) into the DL striatum (Fig. 5c). RM two-way ANOVA revealed significant main effects of treatment (F_(1, 10)_ = 20.9, *P* = 0.0010) and lever (F_(1, 10)_ = 70.2, *P* < 0.0001) and a significant treatment x lever interaction (F_(1, 10)_ = 19.1, *P* = 0.0014) (Fig. 5c). As with ML375, bilateral intra-DL striatal microinjection of varenicline significantly reduced ethanol responding compared to vehicle microinjection (*P* = 0.0003). There was no difference in responding for water after vehicle vs. varenicline microinjection (*P* = 0.96; Tukey’s multiple comparisons test). Interestingly, the effect size for varenicline to reduce ethanol responding was similar to that for ML375 in the same brain region.

Bilateral microinjection of varenicline or vehicle into the DM striatum showed a main effect of lever (F_(1, 15)_ = 84.0, *P* < 0.0001) but no effect of treatment (F_(1, 15)_ = 0.14, *P* = 0.71) (Fig. 5d). Thus, varenicline injection into the DM striatum of iP rats did not affect ethanol responding. Microinjection sites of all rats were validated histologically (Figure S3B).

After identifying the dorsal striatum as a locus for the ability of an M5 NAM to reduce alcohol self-administration, we performed analogous studies with ML380, an M5 positive allosteric modulator (PAM) [17, 34]. We injected a dose of 165 pmol/hemisphere based on in vitro characterisation data and solubility. ML380 had no effect on responding for ethanol in either the DL or DM striatum (Figures S4, S5).

## Discussion

Our results provide the first evidence that a selective, central nervous system (CNS)-penetrant, M_5_ mAChR NAM attenuates ethanol self-administration and cue-induced reinstatement of ethanol seeking. Furthermore, we demonstrate a degree of specificity between ethanol and natural rewards, as ML375 administration did not affect sucrose seeking at equivalent doses that reduced ethanol-seeking behaviours. Given that the mAChR system has an established role in cognition and motor function [36–40], we confirmed that ML375 did not impact procedural memory for lever pressing or normal locomotor activity at the doses used in operant paradigms. Furthermore, we identify the DL striatum, but not the DM striatum, as a locus for ML375 effects on ethanol intake in rats with a history of long-term ethanol self-administration. Notably, intra-DL striatal microinjections of ML375 showed similar efficacy to the clinically used drug varenicline in the same brain region, with the same lack of effect of microinjection into the DM striatum. In this context, it is noteworthy also that the effect size for varenicline in the DL striatum of iP rats (our study) was similar to that mediated by the same dose of varenicline microinjected into the nucleus accumbens core [41]. These results are consistent with previous behavioural studies performed with M_5_ mAChR KO mice, which suggested that inhibiting this mAChR subtype may reduce drug-seeking behaviours without affecting processing of natural rewards [11–13].

Since ~90% of individuals relapse within the first year of abstinence from an addictive substance, with most occurring within the first 3 months, relapse has been identified as one of the key challenges to developing novel and effective therapeutics for treating substance abuse disorders [42, 43]. It was therefore significant that ML375 attenuated cue-induced reinstatement of ethanol seeking. It should, however, be noted that ML375 did not abolish the reinstatement response, consistent with the involvement of other factors in this model of relapse. In addition, it is possible that newer generation M_5_ mAChR-targeting compounds with improved physicochemical or pharmacokinetic properties may show greater efficacy to reduce both self-administration and reinstatement. Of relevance here is also the caveats around solubility and selectivity profile of the M_5_ PAM, ML380. Despite this, our data provide compelling evidence for a role of M_5_ mAChRs in ethanol seeking and highlight a potential new therapeutic target worthy of further investigation.

The dorsal striatum, which shows dense M_5_ mAChR expression, is implicated in the control of drug/ethanol taking after extended experience when behaviour may be habitual in nature [44, 45]. Furthermore within the dorsal striatum there is also a relative shift in engagement from DM to DL striatum following long-term access to drug/ethanol [46–48]. Indeed, previous findings show that the role of the DL striatum becomes increasingly important after extended ethanol self-administration, such that specifically targeting this region is sufficient to reduce ethanol-seeking behaviours [47]. Consistent with this literature, we provide evidence that inhibition of M_5_ mAChR signalling in the DL, but not DM, striatum can regulate voluntary ethanol intake following prolonged use. Importantly, we demonstrate comparable effects for ML375 with another cholinergic ligand, varenicline, which has preclinical and clinical efficacy in reducing ethanol-seeking behaviours [20–23]. Collectively, these data suggest that following prolonged ethanol use there is adaptation of striatal cholinergic systems, thereby adding to the growing body of evidence for the therapeutic potential in targeting cholinergic systems for treating AUDs. Moreover, given the unfavourable side-effect profile of varenicline, which in part relates to its non-selective activity at nAChRs, this ligand is often not well tolerated clinically [49]. Conversely, ML375 selectively targets the M_5_ mAChR and given its restricted expression profile, coupled with the ability of allosteric ligands to modulate endogenous signalling in a way that maintains spatial and temporal signalling, there is likely a reduced risk of on-target side effects, although further study in this area is required [17, 18].

M_5_ mAChRs have a discrete expression profile in the CNS and while the exact mechanism of ML375-mediated modulation of the effects of ethanol are unclear, it is likely that M_5_ mAChRs in the midbrain, striatum and/or ventral subiculum are involved [7, 8, 10]. In the midbrain, M_5_ mAChRs are co-expressed with dopamine D_2_ receptors on dopamine-containing neurons; somatodendritic activation of M_5_ mAChRs on dopamine neurons in the SNc can facilitate the release of dopamine in the dorsal striatum [8, 10, 50]. Furthermore, mAChR agonist-induced increases in striatal dopamine are absent in M_5_ mAChR KO mice and electrical stimulation of either the laterodorsal tegmental nucleus or pedunculopontine nucleus, which normally lead to the release of dopamine in the dorsal and ventral striatum, does not cause dopamine release in M_5_ mAChR KO mice [51–54]. Moreover, it has been proposed that M_5_ mAChRs are located on both ‘dopamine-only’ and on ‘dopamine- and glutamate-’ releasing midbrain axonal projections [7]. Taken together with our data, the M_5_ mAChR likely plays an important role in modulating dopamine and glutamate neurotransmission in areas key to reinforcing the motivational effects of drugs of abuse. Nevertheless, further studies are required to determine the exact mechanism of M_5_ mAChR-mediated regulation of sub-cortical dopamine and glutamate as these systems are not yet fully understood [50].

In conclusion, we provide direct evidence that selective attenuation of M_5_ mAChR signalling with a brain penetrant NAM can modulate ethanol seeking in iP rats. Future studies will elucidate the mechanism(s) for these effects and the extent to which targeting M_5_ mAChRs may provide therapeutic benefits for AUDs.

## Electronic supplementary material

Supp Info

Fig S1

Fig S2

Fig S3

Fig S4

Fig S5
