# Supplementary material for: Muscarinic M5 receptors modulate ethanol seeking in rats
Source: Neuropsychopharmacology. 2018 Feb 5;43(7):1510–7. doi: 10.1038/s41386-017-0007-3 (PMC5983544; doi:10.1038/s41386-017-0007-3)
Supplement: Supplementary file 1 — Supp Info [file 41386_2017_7_MOESM1_ESM.docx]

**Figure S1**

To confirm any activity at sigma (σ)-1 or cannabinoid CB_1_ receptors (noted in cross-screening as potential targets for ML375; Gentry *et al*., 2013), ML375 was tested in assays for both targets. Haloperidol, but not ML375, inhibits [^3^H]-(+)-pentazocine binding in mouse brain homogenates (A), suggesting that ML375 has no appreciable affinity for the ơ1 receptor. ML375 was also tested as an agonist and antagonist in an ERK1/2 phosphorylation assay in FlpIn-CHO cells stably expressing the human CB_1_ receptor. Unlike the classical agonist WIN 55212-2, ML375 had no agonist activity in this assay (B). Furthermore, ML375 failed to inhibit WIN 55212-2 (1µM)-stimulated ERK 1/2 phosphorylation, unlike the prototypical CB_1_ receptor antagonist, SR141716A (C). Sigma-1 data are expressed as a percentage of specific [^3^H]-(+)-pentazocine binding; ERK1/2 phosphorylation data are expressed percentage of the response to 10% FBS. Data represent the mean ± S.E.M. of 5-7 independent experiments performed in duplicate or triplicate.

**Figure S2**

ML375 treatment does not affect the latency to first ethanol response in iP rats (*P* = 0.73). However, ML375 treatment extends the latency to first water response as compared to vehicle (*P* = 0.037). ML375 was dosed at 30 mg/kg (p.o.) at 3 h, 11 h and 27 h prior to testing. White bars represent latency to first ethanol response following vehicle treatment, dashed bar represents latency to first ethanol response following ML375 treatment; black bars represent latency to first water response. Data were analysed by repeated measures two-way ANOVA with post-hoc Tukey’s multiple comparisons test and expressed as mean ± S.E.M.* *P* < 0.05 denotes significant difference of treatment on latency to first water response for ethanol trained rats as compared to vehicle treatment.

**Figure S3**

Neuroanatomical validation of injection sites for (A) ML375- and (B) varenicline- treated rats. Black circles represent DL striatum injections; grey triangles represent DM striatum injections.

**Figure S4**

Bilateral infusion of the M_5_ mAChR PAM ML380 (A; 165 pmol/hemisphere; n = 9; P = 0.82) into dorsolateral [DL] striatum or the dorsomedial [DM] striatum (B; n = 10; P = 0.99) did not impact upon operant self-administration of 10% ethanol in iP rats. White bars represent ethanol responses following vehicle; arrowed bars represent ethanol responses following ML380 treatment; black bars represent water responses. Data are expressed as mean ± S.E.M and were analysed by repeated measures two-way ANOVA.

**Figure S5**

Neuroanatomical validation of injection sites for ML380 - treated rats. Black circles represent DL striatum injections; grey triangles represent DM striatum injections.
