# Supplementary figures and images for: Muscarinic M5 receptors modulate ethanol seeking in rats
Source: Neuropsychopharmacology. 2018 Feb 5;43(7):1510–7. doi: 10.1038/s41386-017-0007-3 (PMC5983544; doi:10.1038/s41386-017-0007-3)

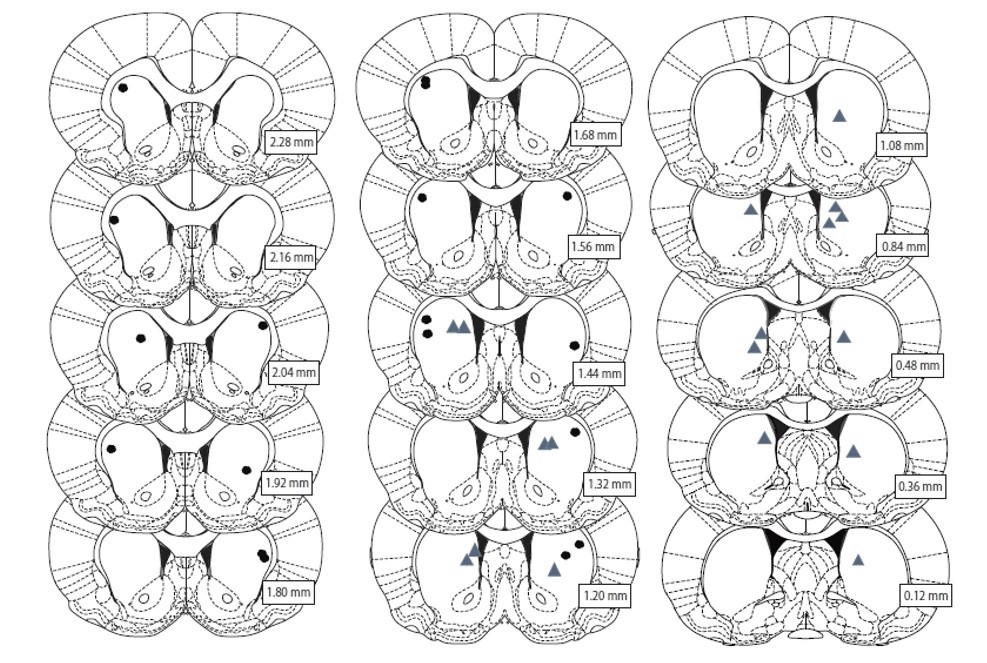

Supplement: Supplementary file 2 — Fig S1 [file 41386_2017_7_MOESM2_ESM.jpg]

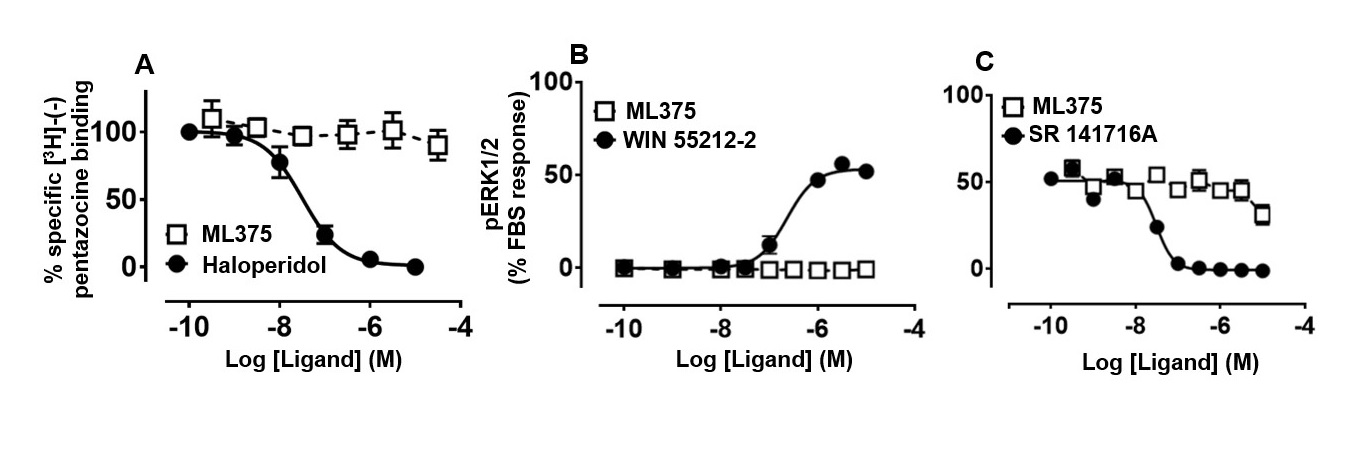

Supplement: Supplementary file 3 — Fig S2 [file 41386_2017_7_MOESM3_ESM.jpg]

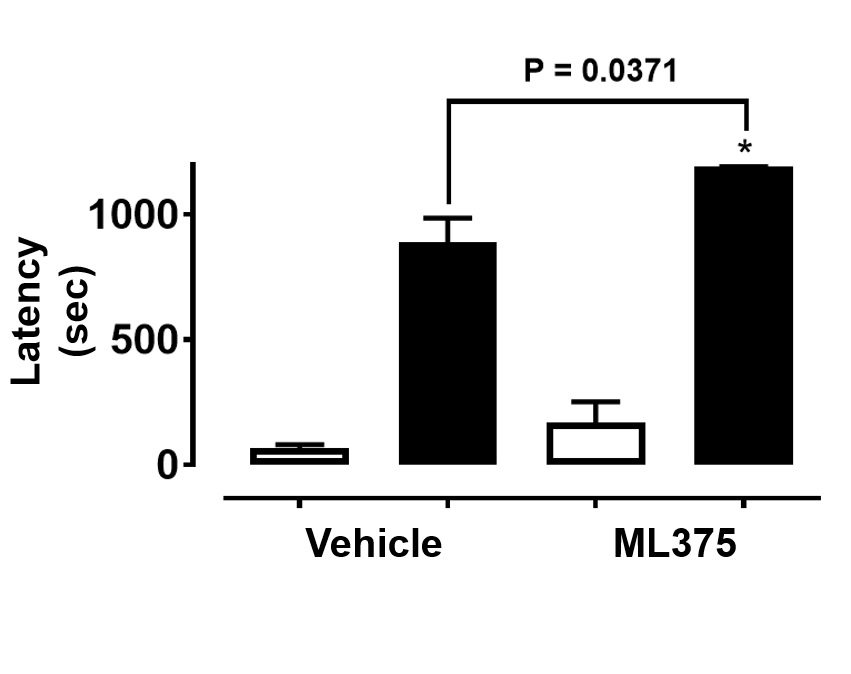

Supplement: Supplementary file 4 — Fig S3 [file 41386_2017_7_MOESM4_ESM.jpg]

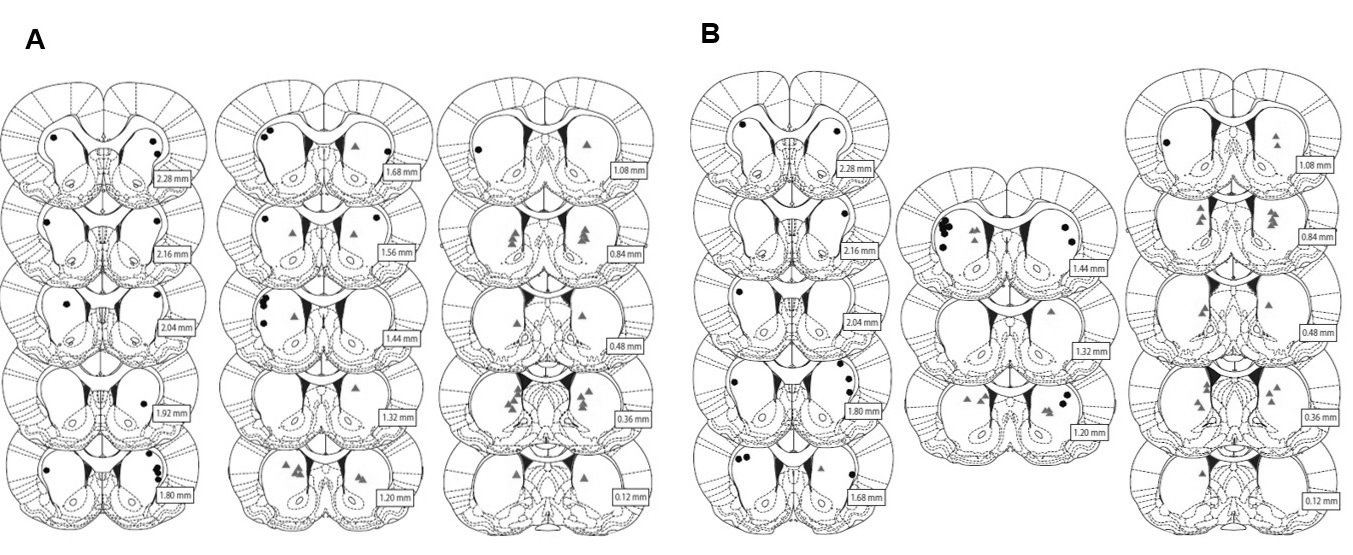

Supplement: Supplementary file 5 — Fig S4 [file 41386_2017_7_MOESM5_ESM.jpg]

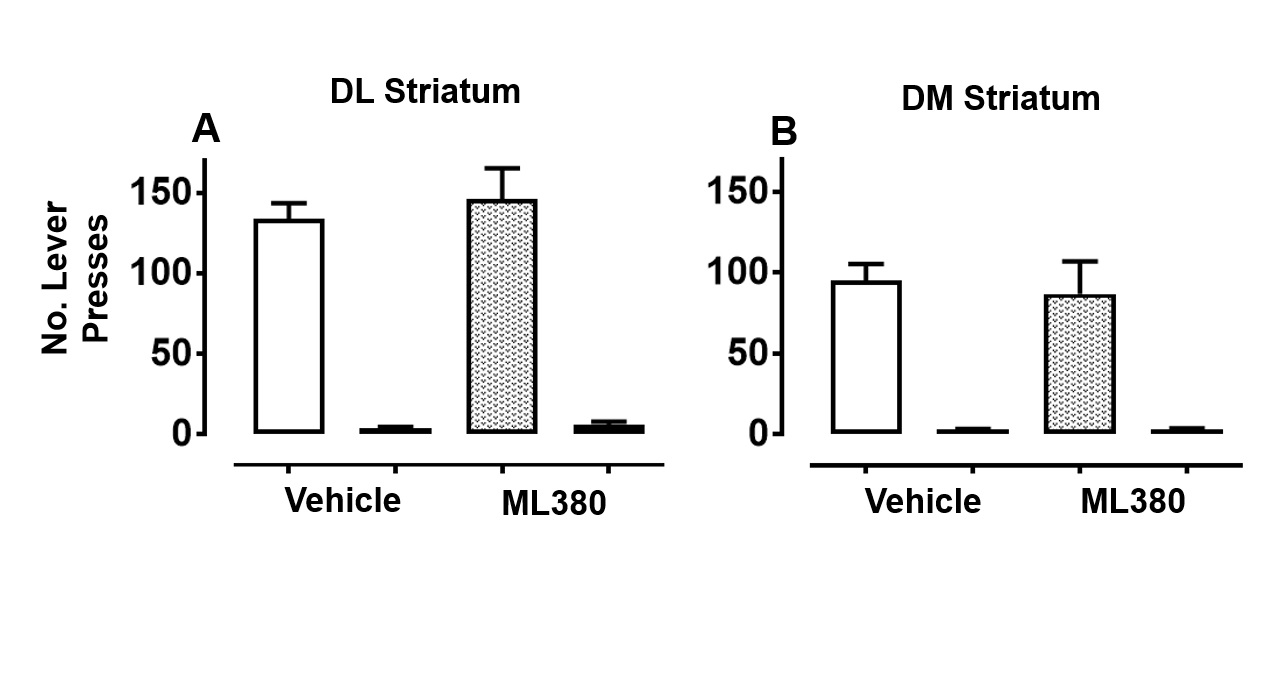

Supplement: Supplementary file 6 — Fig S5 [file 41386_2017_7_MOESM6_ESM.jpg]
